# Generation of diverse neuronal subtypes in cloned populations of stem-like cells

**DOI:** 10.1186/1471-213X-8-89

**Published:** 2008-09-22

**Authors:** Balázs V Varga, Nóra Hádinger, Elen Gócza, Vered Dulberg, Kornél Demeter, Emília Madarász, Balázs Herberth

**Affiliations:** 1 Laboratory of Cellular and Developmental Neurobiology, Institute of Experimental Medicine of the Hungarian Academy of Sciences, Budapest; Hungary; 2 Semmelweis University, Doctoral School, Budapest, Hungary; 3 Agricultural Biotechnology Center, Gödöllő; Hungary; 4 IBDML, CNRS UMR 6216, Campus de Luminy case 907 13288 Marseille Cedex 09 France

## Abstract

**Background:**

The central nervous tissue contains diverse subtypes of neurons with characteristic morphological and physiological features and different neurotransmitter phenotypes. The generation of neurons with defined neurotransmitter phenotypes seems to be governed by factors differently expressed along the anterior-posterior and dorsal-ventral body axes. The mechanisms of the cell-type determination, however, are poorly understood. Selected neuronal phenotypes had been generated from embryonic stem (ES) cells, but similar results were not obtained on more restricted neural stem cells, presumably due to the lack of homogeneous neural stem cell populations as a starting material.

**Results:**

In the presented work, the establishment of different neurotransmitter phenotypes was investigated in the course of *in vitro *induced neural differentiation of a one-cell derived neuroectodermal cell line, in conjunction with the activation of various region-specific genes. For comparison, similar studies were carried out on the R1 embryonic stem (ES) and P19 multipotent embryonic carcinoma (EC) cells. In response to a short treatment with all-trans retinoic acid, all cell lines gave rise to neurons and astrocytes. Non-induced neural stem cells and self-renewing cells persisting in differentiated cultures, expressed "stemness genes" along with early embryonic anterior-dorsal positional genes, but did not express the investigated CNS region-specific genes. In differentiating stem-like cell populations, on the other hand, different region-specific genes, those expressed in non-overlapping regions along the body axes were activated. The potential for diverse regional specifications was induced in parallel with the initiation of neural tissue-type differentiation. In accordance with the wide regional specification potential, neurons with different neurotransmitter phenotypes developed. Mechanisms inherent to one-cell derived neural stem cell populations were sufficient to establish glutamatergic and GABAergic neuronal phenotypes but failed to manifest cathecolaminergic neurons.

**Conclusion:**

The data indicate that genes involved in positional determination are activated along with pro-neuronal genes in conditions excluding any outside influences. Interactions among progenies of one cell derived neural stem cells are sufficient for the activation of diverse region specific genes and initiate different routes of neuronal specification.

## Background

In the course of neural tissue genesis, multipotent neuroepithelial stem/progenitor cells give rise to a number of transient and persisting cell types. Despite the apparent histological homogeneity, the early neuroepithelium is composed of clusters of cell populations with different expression of "pre-neural" and "positional" genes [1,2]. In the closing neural tube, regionalization and segmentation take place well before the formation of neurons [3-5]. The regional heterogeneity is then translated into neuronal sub-type heterogeneity during neural tissue formation [6,7].

The place of origin highly determines the morphology and the neurotransmitter phenotype of future neurons [8,9]. In neuronal progenitors and early precursors, regional determination is thought to decide on the expression of possible homologues of basic helix-loop-helix (bHLH) proneural transcription factors [10,11]. In the forebrain, mash1 bHLH proneural factor is expressed in ventral areas, together with the dlx2 positional factor, which is responsible for the activation of the gad65, a key enzyme of GABA synthesis. The gene cascade is required for the differentiation and migration of ventral forebrain derived GABAergic interneurons of the cortex [12,11,13]. In establishment of the cortical glutamatergic neuronal phenotype, Ngn2, another bHLH proneural factor plays an essential role [12]. Emx2 transcription factor, which is expressed in an overlapping domain with ngn2 helps to maintain the stem/progenitor pool in the ventricular zone of the dorsal anterior forebrain, therefore it appears to be an important regulator of the size of the cerebral cortex [14]. Despite of rapidly accumulating data, the mechanisms underlying the timing and spatial restrictions of the activation of region-specific and proneural genes are not fully understood. Consequently, the regulation of neuronal sub-type determination is not clear.

Because of the complexity of the developing central nervous tissue, simplified *in vitro *models, including cultures of embryonic [15] or adult [16] neuroectodermal progenitors and neurosphere preparations [17,6,18] have been used to investigate neural cell fate decision. The in vitro results indicated some flexible regional commitment of neural stem cells [18,19] and a decisive role of the extracellular environment in regional specification [20,21]. Primary cultures and neurosphere preparations, however, contain multiple types of cells with heterogeneity in both origin and differentiation phase [17]. The heterogeneity hinders the identification of the routes and consequently, the regulatory mechanisms leading to regional and phenotypic determination. Homogeneous starting material for such studies is provided by pheno- and genotypically homogeneous, cloned stem cell populations.

In the presented work, the expression of proneural bHLH transcription factors were investigated in conjunction with the activation of various region-specific genes in the course of in vitro neuron formation by three different, mouse stem-like cell clones including NE-4C embryonic neuroectodermal [22], R1 embryonic stem (ES) [23] and the P19 embryonic carcinoma (EC) [24] cell clones.

The NE-4C clone [22,25] was derived from anterior brain vesicles of p53-deficient early (E9) mouse embryos, and is composed by a nestin expressing population of proliferating neuroectodermal stem cells. If induced by all-trans retinoic acid (RA) or by the presence of perinatal astrocytes [25], NE-4C cells give rise to neurons and later to astrocytes on a highly reproducible schedule [22,26,26]. As a further indication of neural stem cell nature, NE-4C cells integrate into the embryonic brain tissue and provide neurons for the early developing host brain [21]. Implantation-experiments [27] demonstrated that NE-4C cells, *in vivo*, can generate myelin oligodendrocyte-specific protein (MOSP), O1–O4 and RIP immunoreactive oligodendrocytes, as well.

The lack of p53 did not interfere with neuron formation [28,22,26]. Data on the interference of p53 with the activation of region-specific genes, or with the determination of the body axes, however, are missing. Therefore we conducted experiments on two other clones of embryonic stem-like cells (the R1 (ES) [23] and the P19 [24] (EC) cells, both known to have no defect in their p53 pathway. Embryonic stem (ES) and embryonic carcinoma (EC) cell lines represent multipotent populations of early embryonic cells, which can be directed to neural differentiation [29,24,31], but can produce non-neural tissue-type cells, as well. In contrast, NE-4C cells were shown [32] to produce only neural tissue-type cells.

Non-induced cells of each line expressed "stemness genes" along with the early embryonic anterior-dorsal Otx2 gene, but did not transcribe any other investigated CNS region-specific genes. In the course of in vitro induced differentiation, however, several region-specific and diverse sub-type specific pro-neuronal genes were activated. In the absence of any exogenous inducers, cell-autonomous mechanisms were sufficient to drive the manifestation of distinct neurotransmitter phenotypes.

## Results

### In vitro neuron formation by stem-like cells

**Figure 1:**
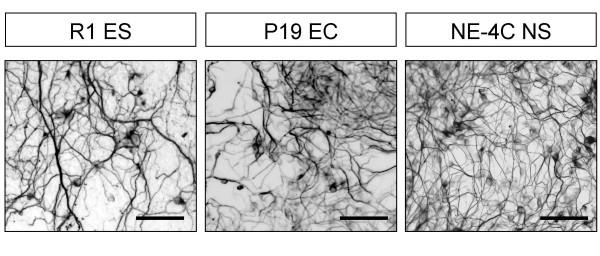
**Neurons develop from ES, EC and NE-4C cells in response to treatment with retinoic acid**. Cultures were stained for neuron-specific βIII-tubulin in a phase of differentiation corresponding to stage 4 of NE-4C development: the 15^th ^day of induced differentiation of R1 (ES), the 4^th ^day for P19 (EC) and the 7^th ^day for NE-4C. Bars: 50 μm

Morphological, cell biological and molecular changes were followed during retinoic acid (*all*-*trans *retinoic acid; RA) induced neural differentiation of R1(ES) [23], P19 (EC) [29,24,30]; and NE-4C embryonic neuroectodermal [22] stem cells. Large number of neurons was formed by all stem-like cell populations (Fig. 1) on a reproducible but cell line-dependent schedule. Bulk neuron formation was evident in P19 cultures by the 3^rd ^day of RA-induction. For mass appearance of neurons, ES cells must have been first grown as aggregates and treated with RA for 4 days (EB4; see M&M), and then incubated in monolayer cultures for a further 7 days.

**Figure 2:**
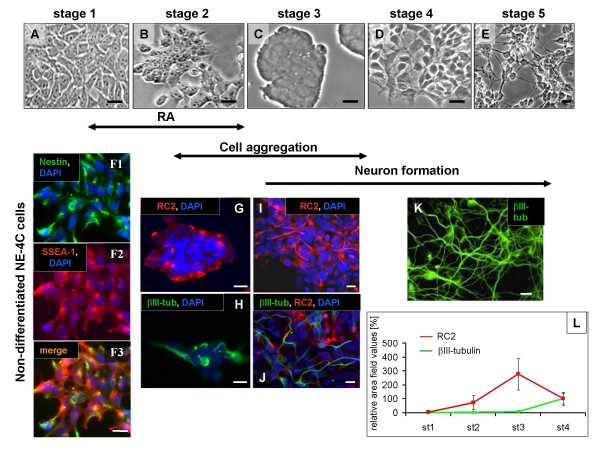
**Morphological and immunocytochemical characteristics of NE-4C cells at defined stages of RA induced neural differentiation**. **A-E**: Phase contrast views of differentiating NE-4C cultures from stage 1 to stage 5. Non-induced (stage 1) NE-4C cells show epithel-like morphology **(A) **and immune-reactivity for nestin and SSEA-1 **(F1–F3)**. RC2 immune-positive cells appear at stage 2 **(B)**, and their frequency increases with formation and compaction of the aggregates **(C, G) **at stage 3. The first βIII-tubulin-positive neuronal precursors **(H) **appear inside the compact aggregates (C). At stage 4, cells migrate out from the aggregates (**D**), and form a two-layer arrangement with a basal substrate-attached monolayer and loose neuronal aggregates on the top of it **(J)**. Morphologically "mature" neurons appear by the end of the first week of induction (stage 5) and form dense networks on the surface of a substrate-attached monolayer **(E, K)**. By the time neurogenesis reaches its highest level, the number of RC2 immune-positive radial-glia like cells decreases **(L)**. Averages of fluorescence area-values for RC2 and βIII-tubulin (see M&M) staining were related to the DAPI-stained area-value representing all cell nuclei, on the same microscopic field. Data are presented as percentages of the related area-values obtained from stage 4 cultures (100%; n = 4). Bars: 10 μm

**Figure 3:**
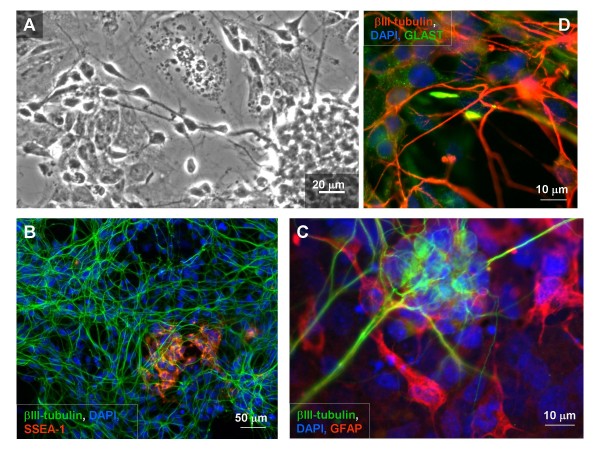
**Differentiated (stage 6) NE-4C cultures contain neurons and astrocytes, but populations of non-differentiated cells persist**. Phase contrast view (A) shows the arrangement of neurons on the top of a substrate-attached monolayer of non-neuronal cells. SSEA-1 positive non-differentiated cells (red on **B**) are located in the substrate-attached monolayer beneath the network of βIII-tubulin (green) immunoreactive neurons. GFAP positive astroglial cells (red on **C**) appear in the substrate-attached monolayer and inside the loose neuronal aggregates among βIII-tubulin (green) immunoreactive neurons. In stage 6, morphologically mature neurons (D) develop in the vicinity of developing and mature glial cells stained for the GLAST glutamate transporter (green). Bars: 20 μm for A, 50 μm for B and 10 μm for C and D

Differentiation of NE-4C cells proceeded through well-characterized stages (see Table 1; Fig. 2, Fig. 3), each marked by a specific combination of immunocytochemical markers [22], appearance of novel cell physiological features [33] and the formation of specific cell-arrangements [22]. RC2 immune-positive, radial glia-like cells appeared by the end of stage 2 and were located in clusters of morphologically yet homogeneous cells (Fig. 2). The first neuronal precursors with βIII-tubulin and NFL immunoreactivity appeared in stage 3, and a large number of developing neurons formed by stage 5. The number of RC2 radial-glia like cells decreased when neurogenesis reached its highest level. (Fig. 2). In stage 6 (Fig. 3), networks of mature neurons formed [34,35] on the top of a substrate-attached monolayer of flat cells containing a number of GFAP-positive astrocytes. SSEA-1 immune-reactive, self-renewing stem cells with neural inducibility (see below) persisted in differentiated (stage 5–6) NE-4C cultures (Fig. 3), and composed substrate-attached spots among differentiated neurons and GFAP-positive astrocytes.

**Table 1:** Stages of RA induced in vitro neural differentiation of NE-4C embryonic neuroectodermal stem cells

No of stage	Name of stage	Days of induction	Cell biological features	Morphology of the culture	Morphology of characteristic cell types	Markers of characteristic cell types
1.	Non-induced	0	continuous proliferation	monolayer	uniform epithel-like morphology stem cells	Nestin [22], SSEA1

2.	Initial changes	1–2	proliferation; selective sensitivity to depolarization (Herberth et al 2002); Altered adhesivity (Schlett et al 2000; Tárnok et al 2002)	monolayer	uniform epithel-like morphology stem cells	Nestin [22], RC2, SSEA-1

3.	Aggregation	2–4	reduced proliferation (Schlett, Madarász 1997) aggregation; compaction; start of neurogenesis	compact aggregates	stem cells; radial glia-like cells, neuronal precursors	SSEA-1, Nestin, RC2 βIII-tubulin, neurofillament-light [15]

4.	Out-migration	3–7	migration from aggregates; neurogenesis, neurite outgrowth	outgrowth zones; monolayers around loose aggregates	radial glia-like cells; stem cells neuronal precursors	RC2, Nestin SSEA-1, MAP2, NeuN, βIII-tubulin

5	Neuronal maturation	6–11	reduction of neurogenesis; neuronal maturation (Herberth et al 2002; Jelitai et al 2002; 2006); network formation; proliferation of basal cells	neural networks on monolayer of basal cells	neuronal precursors; maturing neurons; stem cells	βIII-tubulin, MAP2, NeuN synaptophysyn, NMDA receptors [34] Nestin, SSEA1

6.	Astroglia genesis	10-	aggregation of neurons; fasciculation of neurites; astroglia genesis; proliferation of basal cells	interconnected neuronal aggregates on the monolayer of basal cells	a variety of differentiated neurons; astrocytes; stem cells	βIII-tubulin, MAP2, NeuN, synaptophysyn, NMDA receptors [34] GFAP, S100β [22] and data not show Nestin, SSEA1

### Region-specific genes got activated along with neural differentiation

**Figure 4:**
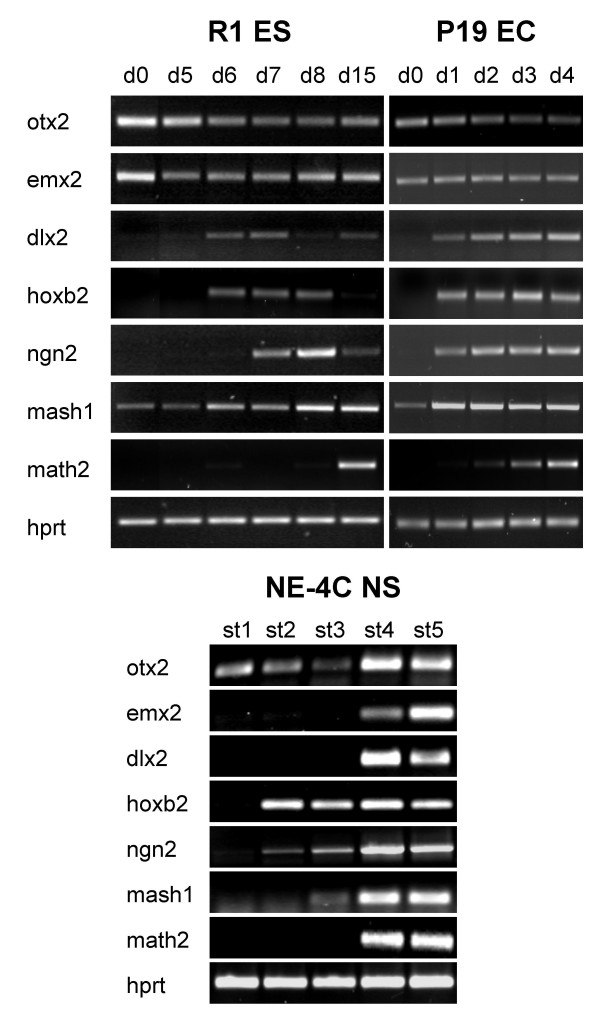
**Activation of region-specific genes is turned on after the onset of neural differentiation**. RT-PCR analyses on the expression of otx2, emx2, dlx2, hox2b region-specific, ngn2, mash1 proneural and math2 neuron-specific genes, in non-induced (d0; st1) and in neuron-enriched (d15, d4, st4) cultures of R1 (ES), P19 (EC) and NE-4C (neural stem) cells, respectively.

Non-differentiated NE-4C stem cells expressed otx2, but no other investigated region-specific factors. Otx2 a known early neuroectodermal marker gene [36] was active also in non-induced ES and EC cells. Non-differentiated R1 ES and P19 EC cells, but not NE-4C cells also expressed emx2 region-specific and mash1 pro-neural genes (Fig. 4).

By the time of the mass-production of neurons displaying βIII-tubulin immune-reactivity and expressing the math2 neuronal gene (see Fig. 1, 2, 3, 4), region-specific genes, emx2, dlx2, hox2b, marking non-overlapping regions in the developing nervous system, were activated in all – ES, EC and NE-4C – cultures (Fig. 4).

**Figure 5:**
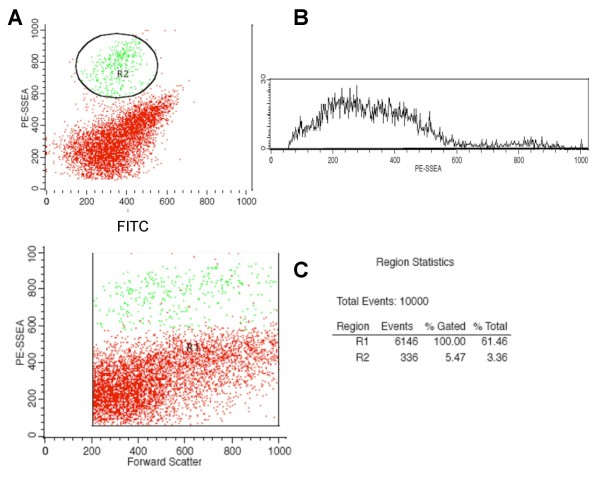
**Flow-cytometric demonstration of SSEA-1 immunoreactive cells (green dots) in differentiated (stage 6) NE-4C cultures**. The dot plot (**A) **shows the SSEA-1 positive population (R2) characterized by high phyco-erythrine (PE) fluorescence (≥ 600; *y*-axis) against a non-specific auto fluorescence (FITC; *x*-axis). The histogram (**B**) and the statistics (**C**) showed an about 5% frequency of SSEA-1 positive cells among the total cells (R1; 100%) defined by forward scatter (FSC) value ≥ 200.

**Figure 6:**
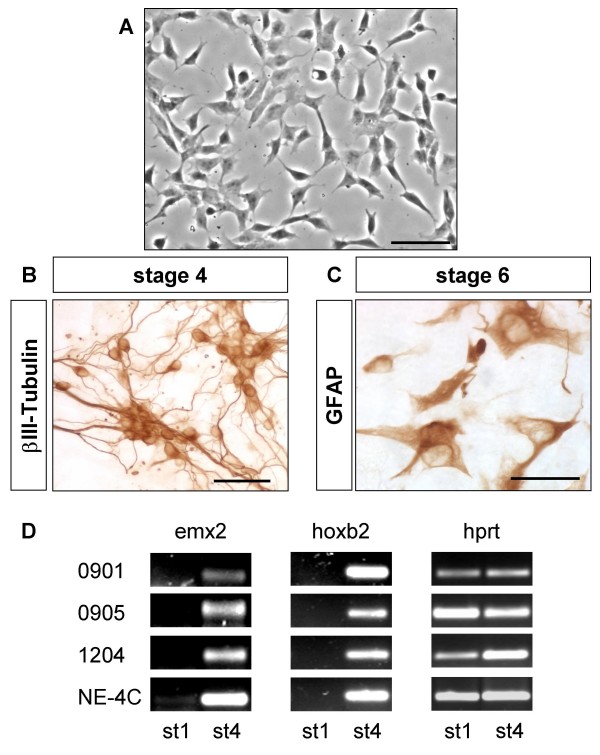
**Non-differentiated, SSEA-1 positive cells re-cloned from differentiated NE-4C cultures display neural stem cell properties**. Non-differentiated cells re-cloned from differentiated (stage 6) cultures displayed epithelial morphology (A) but gave rise to neurons (B) and astrocytes (C) if induced with RA. βIII-tubulin immunoreactive neurons (B) and GFAP-positive astroglial cells (C) visualized by HRP-DAB reaction are shown in a representative clone (clone 0901). Bars: 50 μm on A, C and 100 μm on B. **D**: Investigated sub-clones (clone 0901; 0905; 1204) showed a gene expression pattern characteristic to non-induced (stage1) NE-4C cells, but expressed a variety of region-specific genes after reaching more differentiated stage (st4).

SSEA-1 positive, non-differentiated cells persisting in differentiated NE-4C neural cultures (Fig. 3A) and comprising 2 to 5% of total cells, were separated by FACS sorting for SSEA-1 immunoreactivity (Fig. 5). From the selected SSEA-1 positive cell fraction several one-cell derived colonies were established by cell cloning. These subclones of morphologically non-differentiated (Fig. 6A), proliferating cells could be induced by RA to give rise to neurons and astrocytes (Fig. 6B, C), indicating a preserved developmental capacity of NE-4C mother cells. In their non-induced stage (stage 1), SSEA-1 sorted sub-clones did not express the investigated region-specific genes (Fig. 6D), except otx2 (data not shown). A variety of region-specific genes, however, became activated by the time of appearance of neurons (stage 4). The data indicated that expression of diverse region-specific genes was induced along with neural differentiation.

### Particular region-specific genes become activated in different stages of in vitro neural differentiation

**Figure 7:**
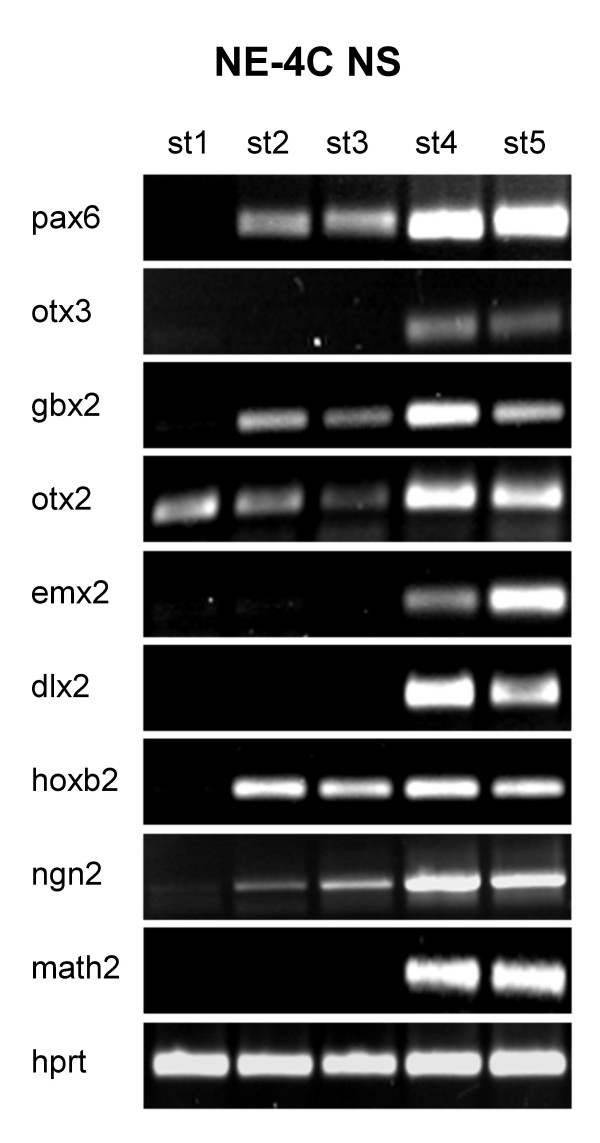
**Expression of region-specific genes in cultures of NE-4C neural stem cells at different stages of neural differentiation**. A group of region-specific genes including pax6, gbx2, and hoxb2 was activated at the time of switching on proneural (e.g. ngn2) genes. The expression of another set of "positional" genes (dlx2, emx2, otx3) was detected in a later phase of differentiation, along with the activation of math2 neuronal gene.

The onset of detectable transcription from the investigated region-specific genes seemed to be bound to two different stages of neural development of NE-4C cells (Fig. 7). One set of region-specific genes including pax6, gbx2 and hoxb2 were activated as early as stage 2, when pro-neural gene-activity had already been turned on. (Interestingly, R1 ES and P19 EC cells expressed mash1 in their non-differentiated stages while another proneural gene ngn2 became activated only later, coinciding with the onset of neuron-morphogenesis (Fig. 4))

The expression of another group of region-specific genes (emx2, dlx2 and otx3) was detected not sooner than stage 4, when math2, a marker for postmitotic maturing neurons [37] was also active (Fig. 7).

**Figure 8:**
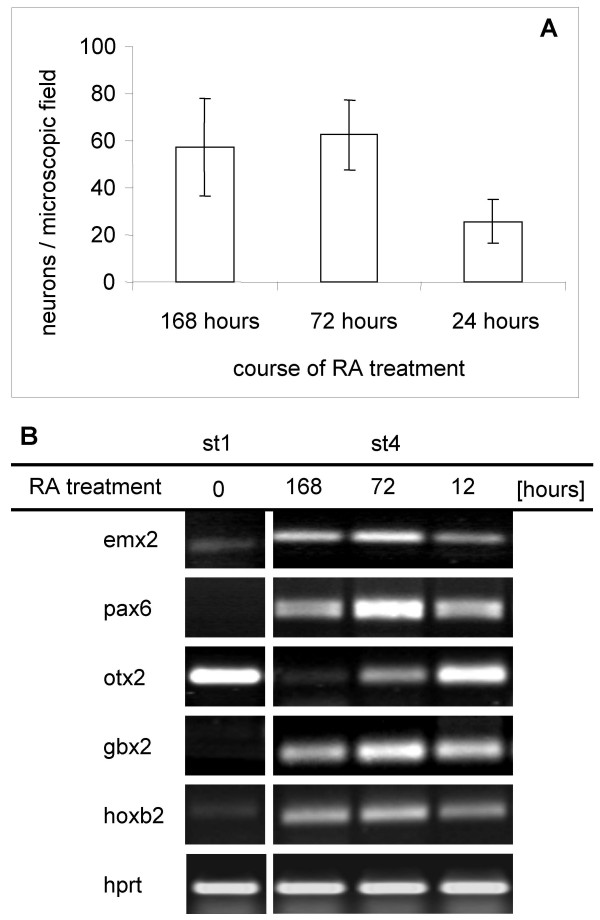
**Neuron formation and gene expression after short- or long-term exposure to RA**. Differentiation of NE-4C cells was induced by exposure to 10^-6 ^M RA for 12, 72 or 168 hours. **A**: The number of neurons was determined by counting NeuN-positive nuclei after a 168-hour period of differentiation (stage 4). In comparison to longer (72-hour or 168-hour) treatments, short term (12-hour) induction resulted in less NeuN-positive neurons. (Averages and standard deviation values were calculated from 4 identically treated sister-cultures (n = 4)). B: Short-term (12-hour) or long-term (72 or 168 hours) presence of RA did not cause significant changes in the expression of hox2b, gbx2 and pax6 genes. The continuous presence of RA did not inhibit the expression of the dorsal forebrain specific emx2 gene, but was sufficient to reduce the RA-sensitive otx2 mRNA level. The results of a representative RT-PCR assay on non-induced (stage 1; left column) and differentiated, neuron-rich (stage 4; three right columns) cultures are shown.

Bearing in mind that RA may also serve as a "posteriorizing and ventralizing" agent [38], we compared the effects of short-and long-term presence of RA. In addition to the standard 48-hour treatment, NE-4C cells were exposed to 10^-6 ^M RA for 12, 72 and 168 hours and the expression of several region-specific genes was investigated on the 7^th ^day (stage 4) of induction (Fig. 8). Each exposure to RA resulted in neuron formation (Fig. 8A), but the proportion of neurons was smaller after the shortest (12 hour) induction. Long-term exposure to RA (168 h for NE-4C and 96 hours for R1 ES) effectively reduced the otx2 transcript level (Fig. 8B), but failed to cause relevant changes in the expression of many positional genes including the anterior-dorsal emx2.

### One-cell derived progenies can acquire diverse neurotransmitter phenotypes

**Figure 9:**
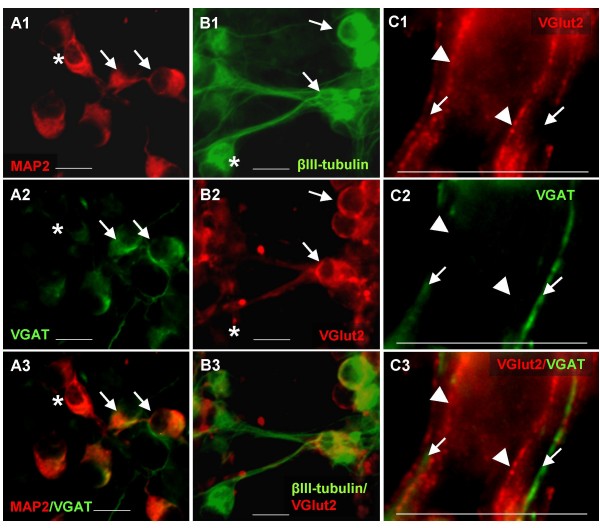
**NE-4C derived neurons can acquire different neurotransmitter phenotypes**. **A**: Double immune-staining for MAP2 (red; A1, A3) neuronal marker and for VGAT (green; A2, A3) GABAergic neuronal marker showed that VGAT staining was confined to neurons, but not all neurons displayed VGAT-immune-reactivity. Arrows mark double immune-reactive cells; asterisk indicates a VGAT-negative neuron. **B**: Double immune-staining for βIII-tubulin (green; B1, B3) and VGLUT2 (red; B2, B3) glutamatergic neuronal marker demonstrated the presence of both glutamatergic (arrows) and non-glutamatergic neurons. Asterisk marks βIII-tubulin positive but VGLUT2 negative neurons. **C**: Double staining for VGAT (green; C2, C3) and VGLUT2 (red; C1, C3) revealed a complete segregation of GABAergic (arrows) and glutamatergic (arrowheads) processes. Immunostaining was made on differentiated NE-4C cultures at stage 6. Bars: 10 μm

The expression of different homologues of proneural genes (ngn2 and mash1) and the activation of diverse region-specific genes suggested a possible diversity in the neurotransmitter phenotype adopted by NE-4C neurons. Immunocytochemical analyses of differentiated (stage 5 – 6) NE-4C cultures demonstrated GABAergic (Fig. 9A) and glutamatergic (Fig. 9B) cells among mature, NeuN [39], MAP2 or βIII-tubulin immunopositive neurons. GABAergic neurons were identified by the presence of the vesicular GABA transporter (VGAT). Glutamatergic neurons were stained for the vesicular glutamate transporter (Vglut2). In stage 6, these two neuronal subtypes, together, composed the majority (approximately 95%) of NeuN positive cells (data not shown).

**Figure 10:**
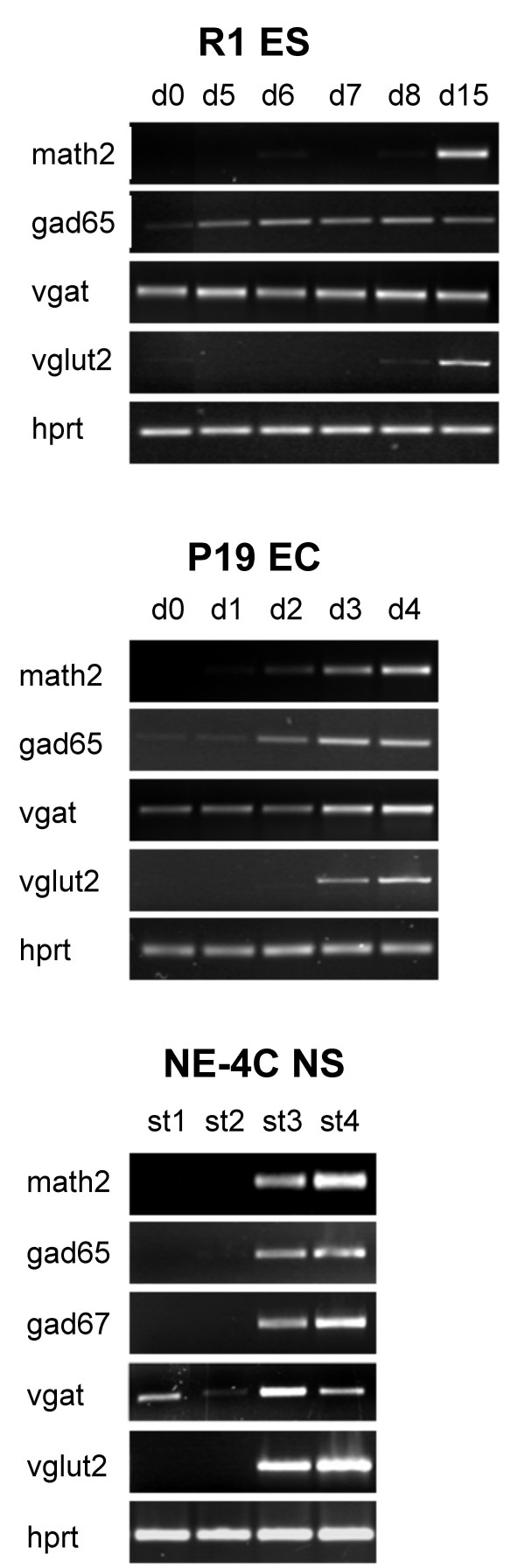
**Expression of genes indicating glutamatergic and GABAergic neuronal phenotypes**. The expression of the GABAergic phenotype-related gad67 or gad65 and the glutamatergic vglut2 genes was detected in math2 expressing, neuron rich cultures of all (R1 ES, P19 EC and NE-4C NS) cell lines. Unexpectedly, vgat was expressed by all types of non-induced stem cells. Samples were taken on different stages of neural differentiation; for R1 ES on days 0,5,6,7,8,15 (see M&M); for P19 EC on days 0, 1, 2, 3, 4 after RA-induction; for NE-4C NS: at stages 1,3,4,5 (see Table 1).

In addition to demonstrating the activity of proneural genes linked to the establishment of the GABAergic and glutamatergic phenotypes (mash1 and ngn2, respectively), several genes involved in the maintenance of these neurotransmitter phenotypes were transcribed in differentiating NE-4C cultures (Fig. 10).

As expected from earlier results [35], many genes related to GABA metabolism were active at early stages of neural differentiation (Fig. 10), well before the appearance of morphologically identified neurons. While Ebihara and colleagues [40] reported on a low-level vgat expression in non-induced P19 cells, the ubiquitous presence of VGAT mRNA in all non-induced stem-like cells, was a rather unexpected finding. In contrast to a marked presence of vgat mRNA in non-induced NE-4C cultures, VGAT immunoreactivity was detected only in a proportion of mature neurons at stages 5 and 6 (Fig. 9A, C). By this stage, genes coding for the GABA synthesizing enzymes (gad65 and gad67) were also active (Fig. 10).

The vesicular glutamate transporter type 2 (Vglut2), was undetectable in non-differentiated stem-like cell cultures, either at mRNA or protein level. The activation of vglut2 gene was detected only in neuron-rich, math2 expressing cultures derived either from ES, EC or from neuroectodermal stem cells (Fig. 10). Accordingly, VGlut2 immunoreactive neurons were detected only in differentiated neuronal cultures from stage 4 onwards (Fig. 9B, C). In order to decide whether individual NE-4C derived neurons were exclusively committed to either GABAergic or glutamatergic fates, neurons were double-stained for VGAT and VGLUT2 proteins (Fig. 9C). Double immunolabeling revealed a complete segregation of VGAT and VGLUT2 staining, indicating the differentiation of at least two distinct subtypes of neurons.

**Figure 11:**
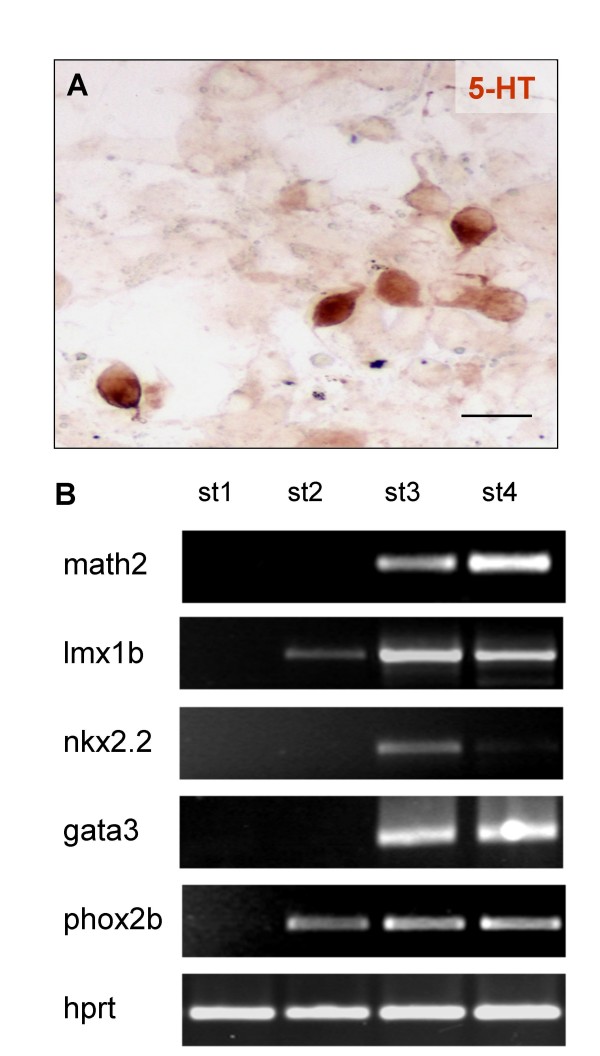
**Monoaminergic features in differentiating NE-4C cultures**. **A**: 5HT staining of NE-4C derived neurons at stage 5, scale bar: 10 μm. Approximately 1% of the neurons contained 5 HT (see also Table 2). B: Several genes coding for transcription factors involved in the differentiation of monoaminergic neurons were transcribed in stage 4 and 5 neuron rich (math2 expressing) cultures.

While a few serotonin containing (Fig. 11A) cells were revealed, NE-4C neurons with tyrosine-hydroxylase immunoreactivity were not found (Table 2). Genes coding for transcription factors involved in the regulation of monoaminergic differentiation (lmx1b, nkx2.2, [41] gata3 [42], phox2b [43], on the other hand, were transcribed by stage 4 (Fig. 11B). According to gene array analysis (see Additional File 1), however, the expression from a number of genes related to monoaminergic development [44,45] did not increase significantly in the course of in vitro induced neuron formation.

**Table 2:** Distribution of investigated neurotransmitter phenotypes in stage 6

**Transmitter phenotype**	**Investigated molecule**	**Percentage of neurons**
GABAergic	GABA, VGAT	57,7 ± 8,2*
Glutamatergic	VGlut2	44.2 ± 1,6*
Cathecolaminergic	TH	Not detected
Serotonergic	5 HT	< 1

100% was determined as the total number of the NeuN positive neurons

The data demonstrated that after initiation of neural differentiation by RA, intrinsic interactions between one-cell derived neural progenitors were sufficient to establish distinct GABA or glutamatergic neurotransmitter phenotypes, but did not result in the formation of catecholaminergic neurons.

## Discussion

In non-induced stages, one-cell derived populations of ES, EC and neuroectodermal stem cells expressed only early anterior-dorsal region-specific genes, characterizing the developing epiblast/neural plate areas [46]. Upon neural differentiation, a variety of region-specific genes, those expressed in non-overlapping regions of the developing CNS, were activated.

The activation of positional genes seemed to depend on the stage of differentiation. The R1 ES, P19 EC and NE-4C neuroectodermal stem cells, in their non-induced states, represented different stages of stem cell differentiation. For neural commitment, ES cells had to be preincubated to form embryoid body like aggregates [47] prior to and during the induction with RA, and mature neurons developed only after a further growth in monolayer cultures. P19 cells, on the other hand, produced neurons in a short period (3 days) after RA-induction even in comparison to the 5–7 days neurogenetic period of NE-4C cells. Despite the above differences, RA-induced neural differentiation could be compared among the different cell lines if the activation of ngn2 proneural gene was set as a reference point (3^rd ^day for ES, 1^st ^day for P19 and 2^nd ^day for NE-4C after the onset of RA induction).

In response to RA-induction, ES and EC cell populations could give rise to a number of non-neural cells in addition to the differentiation of neurons [48,49]. In such preparations, neural and non-neural cells could interact with each other, mimicking some in vivo interplay between the developing neural and non-neural tissues. In the case of NE-4C neuroectodermal cells, which do not produce any non-neural progenies [32] different region-specific genes were activated by mechanisms inherent to the one-cell derived neuroectodermal population.

A set of region-specific genes, including gbx2, hoxb2 and pax6 became active along with pro-neural genes, at a stage when the RC2 immunoreactive radial glia-like phenotype appeared in induced NE-4C cultures. In case of the hox-family genes, known to respond directly to RA [4,50], rapid gene activation was expected in response to RA. Direct RA-responsiveness of gbx2 or pax6 genes, however, was not reported. Another set of "positional" genes, including emx2, otx3, dlx2 nkx2.2, lmx1b, gata3, expression was detected only after the activation of the neuron-specific math2 gene. The delayed activation of these genes could not be the result of any direct effect of RA, as RA was removed days before the onset of the gene activation.

The applied conditions were sufficient to induce and maintain GABAergic and glutamatergic fates in NE-4C neurons, but failed to manifest catecholaminergic or cholinergic (data not shown) features. The formation of catecholaminergic neurons from ES cells [51], requires soluble factors such as SHH, FGF2 and FGF8. RA was reported to promote the production of SHH and FGF8 in the developing chicken brain [52], but SHH production was demonstrated in quails even at complete lack of RA [53]. According to gene array analyses (see Additional File 1), transcription from fgf8 and shh did not change in the course of neural differentiation of NE-4C cells. The activation of genes indicating catecholaminergic development including nurr1 [54] or tyrosine-hydroxylase (th), showed either a short transient increase or did not change in the course of neuron formation by NE-4C cells. The absence of catecholaminergic cells suggest an insufficient level of factors necessary for the establishment of monoaminergic phenotypes [44]. Future experimentation will determine whether the few serotonin containing neurons represented a serotonergic population or only a subpopulation of GABAergic neurons containing 5 HT as a neuromodulator [55].

In our experiments, RA was withdrawn from the environment of differentiating cells after initiation of neural differentiation. The intrinsic interactions in differentiating populations of one cell derived NE-4C cells could result in the production of glutamatergic and GABAergic neurons, but failed to manifest all of the major neurotransmitter phenotypes. While several posterior (hoxb2, gbx2, phox2b, lmxb1) and ventral (dlx2) positional genes were activated, this activity was insufficient to manifest catecholaminergic phenotypes in NE-4C derived neurons. It may be due to the restricted developmental potential of the cell line, or may indicate a requirement of extrinsic factors for cholinergic or catecholaminergic commitment.

## Conclusion

The data demonstrated that cell-autonomous mechanisms can direct diverse "regional" specifications in positionally non-determined neuroectodermal cells and manifest different – at least GABAergic and glutamatergic – neurotransmitter phenotypes.

## Methods

### Maintenance and neural induction of NE-4C and P 19 cells

The NE-4C and P19 cells were maintained in Minimum Essential Medium (MEM; Sigma) supplemented with 5% fetal calf serum (FCS; Gibco), 4 mM glutamine (Sigma) and 40 μg/ml gentamycin (Chinoin), at 37°C temperature with 5% CO_2_. Sub-confluent cultures were regularly split by trypsinisation using 0.05% trypsin (Sigma) in phosphate-buffered saline (PBS) and transferred into poly-L-lysine (PLL) (Sigma) coated dishes at a cell density of 10^4 ^cells/cm^2^. Neural differentiation was induced with a 48-hour treatment with 10^-6 ^M *all-trans *retinoic acid (RA) (Sigma). The medium was changed every 2^nd ^day.

### Isolation of stem cells from differentiated NE-4C cultures

Cells were harvested from differentiated cultures by mild agitation in 1 mM EDTA in PBS. For fluorescent activated SSEA-1 cell sorting, the cell suspension was incubated with a pre-established immuno-complex (mixed in 1 to 1 v/v for 30 min at room temperature) of SSEA-1 (1:2000; DSHB) and anti-mouse IgM conjugated-phycoerythrin (1:500; BD-Pharmingen) antibodies. For fluorescent background control we used cells treated only with anti-mouse IgM conjugated-phycoerythrin antibody. Cytofluorimetric analyses and cell sorting was done with FACS Vantage (BD) instrument at a flow rate of 1000 events/sec. Sorted cells were seeded into PLL-coated dishes at low cell densities. Colonies derived from single cells were isolated by cloning rings. Sorted SSEA-1 positive cells or cells obtained from cloning rings were grown in standard medium. The neural inducibility was checked by treatment with RA according to the standard induction protocol.

### ES cell culture and differentiation

R1 [23] ES cells were maintained on a feeder layer of embryonic mouse fibroblasts, in ES cultivation medium composed of Dulbecco's modified Eagle's medium (KO-DMEM medium; Gibco) supplemented with glutamax (Gibco, 100×), 50 μg/ml streptomycin (Sigma), 50 U/ml penicillin (Sigma), 50 mM β-mercaptoethanol (Sigma), 0.1 mM non-essential amino acids (Gibco), 1000 units/ml of leukemia inhibitory factor (Esgro) and 20% FCS (HyClone).

Two days before induction, ES cells were transferred to gelatin (Sigma) coated Petri dishes (Greiner) for 24 hours (day 0 of differentiation), then harvested and transferred into bacteriological dishes in differentiation medium (KO-DMEM supplemented with glutamax, 50 μg/ml streptomycin, 50 U/ml penicillin, 50 mM β-mercaptoethanol, 0.1 mM non-essential amino acids, and 15% FCS) (5 × 10^6 ^cells/10 ml). Embryoid bodies (EBs) were formed in suspension were transferred into fresh dishes with fresh medium every second day. *All-trans *retinoic acid (10^-6 ^M; Sigma) was added for 4 days, between the 4th and 8th days. On the 8th day of differentiation, EBs were plated onto gelatin-coated plates and maintained for a further 7 days.

### RT-PCR analysis

NE-4C, R1 (ES) and P19 (EC) cells were lysed by addition of Tri Reagent (Sigma) according to the instruction of the manufacturer. Total RNA fraction was then isolated using organic/inorganic extraction by the standard procedures. DNA contamination was eliminated by DNase-I (Fermentas) treatment. The isolated RNA was suspended in RNase/DNase free water at a concentration of 1 μg/μl and stored at -70°C. Reverse transcription (RT) reactions were undertaken from 1.5 μg total RNA using First strand cDNA synthesis Kit (Fermentas) at 42°C. The quantity and a potential genomic DNA contamination of the cDNA product was determined by PCR (Hotstart Taq PCR Kit (Qiagen)) using primers recognizing both cDNA (248 bp) and genomic DNA (1086 bp) sequences of the house keeping hypoxanthine guanine phosphoribosyl transferase coding gene (hprt). cDNA samples containing no genomic DNA were diluted according to the amplified hprt product and used for further PCR analyses. The optimum temperatures, cycle numbers and MgCl_2 _concentrations were set for each primer pairs (Table 3). The PCR products were run in ethidium bromide containing agarose gels, and were visualized by UV trans-illumination.

**Table 3:** Primers used for RT-PCR analysis

Gene symbol	Forward	Reverse
dlx2	5'cagggtccttggtctcttca3'	5'ctgctgaggtcactgctacg3'
otx3	5'gcagctccagaaacagaa3'	5'gagtcctcttcacggtccag3'
emx2	5' gtcccagcttttaagctaga3'	5'cttttgccttttgaatttcgttc3'
gad65	5'ggtctggcttttggtccttc3'	5'tgccaattcccaattatactcttga3'
gad67	5'gctggaaggcatggaaggtttta3'	5'tgagccccatcaccgtagca3'
gata3	5'acgtctcactccgaggcagcatg3'	5'gaagtcctccagcgccgtcatgcac3'
gbx2	5'ttcgaagtcaacaccagcag3'	5'cccctttaagcccgtctaat3'
hoxb2	5' gctggagaaggagttccact3'	5'tagggaaactgcaagtcgat3'
hprt	5' cacaggactagaacacctgc3'	5'gctggtgaaaaggacctct3'
lmx1b	5' tggagtagagccggtcaatg3'	5'ctgatgcgagtcaacgagtc3'
mash1	5'ttagtccagaggaacaagagctgc3'	5'aagatgcaggatctgctgccatcc3'
math2	5'tgagaatggcttgtccagaagg3'	5'tggtagggtgggtagaatgtgg3'
ngn2	5'aagaggactatggcgtgtgg3'	5'atgaagcaatcctccctcct3'
nkx2.2	5'ggttccagaaccatcgctac3'	5'caccgaaaacaaacgacaaa3'
otx2	5'ggaaacagcgaagggagagga3'	5'ctgctgctgttggcggcactt3'
pax6	5'acgaaagagaggatgcctc3'	5'cccaagcaaagatggaag3'
phox2b	5'ctcctacccctttcctcacc3'	5'cagctcggatcactcaatca3'
vgat	5'acgaggagaacgaagacgg 3'	5'acgatgatgccaatggagat 3'
vglut2	5'tggaaaatccctcggacaga3'	5'tagacgggcatggatgtgaa3'

Total RNA fractions obtained from NE-4C cultures at various (1, 2, 3, 4, 6) stages of in vitro neural differentiation were analyzed also by AGILENT Mouse Developmental RNA microarray (Kromat Ltd, Budapest, Hungary) assays (see Additional File 1).

### Immunocytochemical staining

Cultures were fixed with 4% paraformaldehyde in PBS at room temperature for 20 min and the cells were treated with 0.1% Triton X-100 (Promega) for 60 min for NeuN detection and 10 min for all others. After rinsing, non-specific antibody binding was blocked by incubating with 5% FCS in PBS (PBS-FCS) for 1 hr. Primary antibodies were diluted in PBS-FCS. Antibodies to SSEA-1 (DSHB), RC2 (DSHB), βIII-tubulin (ExBio Praha), GFAP (Sigma), VGlut2 (Synaptic Systems), GABA (Sigma), GLAST (4ADI) and NeuN (Chemicon) were diluted to 1:1000. Anti-MAP2 (Chemicon), a-VGAT (Synaptic Systems) and a-serotonin were used in 1:100. The fixed cultures were incubated with the primary antibodies at 4°C, overnight. Alexa-488 and Alexa-594 conjugated secondary antibodies (Molecular Probes) and biotin-conjugated secondary antibodies (Vector) were used at 1:1000 dilution, for 60 min at room temperature. Streptavidin-conjugated Alexa-488 and Alexa-594 (1:1000) (Molecular Probes) were added for 60 min at room temperature. Pictures were taken with a Zeiss Axiovert 200 M microscope.

### Calculation of the proportion of different cell types

Total RC2 and βIII-tubulin immunoreactivities were determined by measuring the fluorescent areas using Axiovision 4.5 software. 30 microscopic fields (10 × objective) from each developmental stages from 4 independent experiment series were measured. Values of field-fluorescence were averaged and standard deviations were calculated. Relative area-values have been calculated as percentages of the values obtained from cultures at developmental stage 4 (100%) (for developmental stages see Table 1.)

The total number of mature neurons was determined by counting NeuN – positive cells on the 12^th ^day (stage 6) of RA-induction. NeuN-positive cells, also immunoreactive for GABA or VGAT were regarded as GABAergic neurons. Those displaying Vglut2 and NeuN double-staining were counted as glutamatergic neurons. Immunopositive cells were counted at 400× magnification. The means and standard deviations were calculated from at least 12 microscope fields from each of three equally treated sister cultures (n = 36). The number of GABAergic glutamatergic and serotonin-containing neurons has been presented as percentages of the total number of NeuN-positive cells (100%).

## Abbreviations

5 HT: Serotonin; bHLH: Basic Helix Loop Helix transcription factor; CNS: central nervous system; dlx2: Distal-less homeobox 2; EC: Embryonic carcinoma; emx2: Empty spiracles homolog 2; ES embryonic stem cell; GABA: Gamma amino butyric acid; gad65: Glutamic acid decarboxylase gene for isoform 65 kDa; gad67: Glutamic acid decarboxylase gene for isoform 67 kDa; gata3: GATA binding protein 3; gbx2: Gastrulation brain homeobox 2; GFAP: Glial fibrillary acidic protein; hoxb2: Homeo box B2; hprt: Hypoxanthine guanine phosphoribosyl transferase; lmx1b: LIM homeobox transcription factor b1; mash1: Mouse achaete-scute complex homolog-like 1; math2: Mouse atonal homologue 2; ngn2: Neurogenin-2; nkx2.2: Nk2 transcription factor related locus 2; otx2: Orthodenticle homologue 2; otx3: Orthodenticle homologue 3; pax6: Paired box 6; phox2b: Paired-like homeobox 2b; RA: Retinoic-acid; SSEA1: Stage specific embryonic antigen-1; TH: Tyrosine-hydroxylase; VGAT: Vesicular GABA transporter; VGLUT2: Vesicular glutamate transporter type 2,

## Authors' contributions

B.V. designed and carried out molecular biological experiments, contributed to constructing the manuscript.

N.H. carried out cell culture experiments, immunocytochemical staining, RT-PCR analyses

V.D. cloned and analyzed self-renewing sub-clones

K.D. carried out flow-cytomtery analysis and sorting of cells.

E.G. cultivated and differentiated mouse ES (R1) cells; provided ES samples for molecular analyses

E.M. contributed to designing the experiments, to analyzing and interpreting the data and to writing the manuscript

B.H. designed experiments, coordinated the experimental work, contributed to constructing the manuscript.

All authors read and approved the final manuscript.

## Supplementary Material

Additional file 1 **RNA microarray analysis of monoaminergic neuronal phenotype related genes.** Expression level of monoaminergic neuronal phenotype related genes (th, bmp2, bmp4, fgf8, fox2a, gata3, pax5, hand2, msx1, nurr1, shh) were analysed in samples from NE-4C cultures in different stages of neural differentiation. Gene expression levels of emx2, gbx2, mash1 and gata3, that were analysed with RT-PCR method and presented in the results section, are shown here also for comparison. Click here for file
