# Supplementary material for: Generation of diverse neuronal subtypes in cloned populations of stem-like cells
Source: BMC Dev Biol. 2008 Sep 22;8:89. doi: 10.1186/1471-213X-8-89 (PMC2556672; doi:10.1186/1471-213X-8-89)
Supplement: Additional file 1 — RNA microarray analysis of monoaminergic neuronal phenotype related genes. Expression level of monoaminergic neuronal phenotype related genes (th, bmp2, bmp4, fgf8, fox2a, gata3, pax5, hand2, msx1, nurr1, shh) were analysed in samples from NE-4C cultures in different stages of neural differentiation. Gene expression levels of emx2, gbx2, mash1 and gata3, that were analysed with RT-PCR method and presented in the results section, are shown here also for comparison. [file 1471-213X-8-89-S1.pdf]

Samples were collected from stage 1 (st1), 2 (st2), 3 (st3), 4 (st4) cultures of NE-4C cells. The RNA integrity was first examined with an Agilent 2100 Bioanalyzer. The generation of cDNA, production of labelled cRNA, and hybridization to Agilent G4120A Mouse Development Microarrays were performed according to standard protocols provided by the manufacturer at the Microarray Core Facility at the Department of Genetics, Cell- and Immunobiology at Semmelweis University (Budapest, Hungary). All materials are from Agilent (Agilent, Santa Clara, CA 95051

United States) except RNA isolation kit from Qiagen.

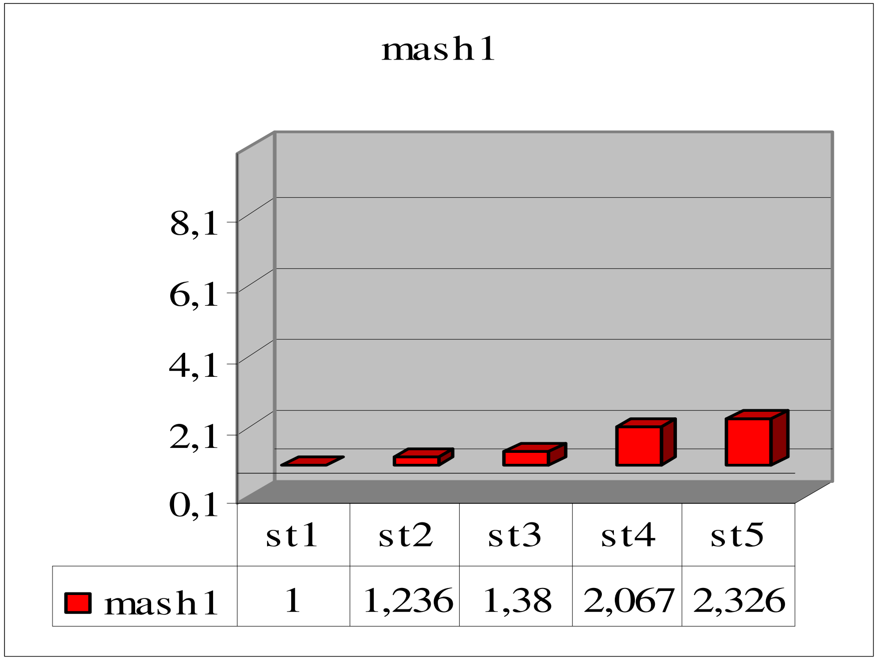
mash1
8,1
6,1
4,1
2,1
0,1
st1
st2
st3
st4
st5
mash1
1
1,236
1,38
2,067
2,326

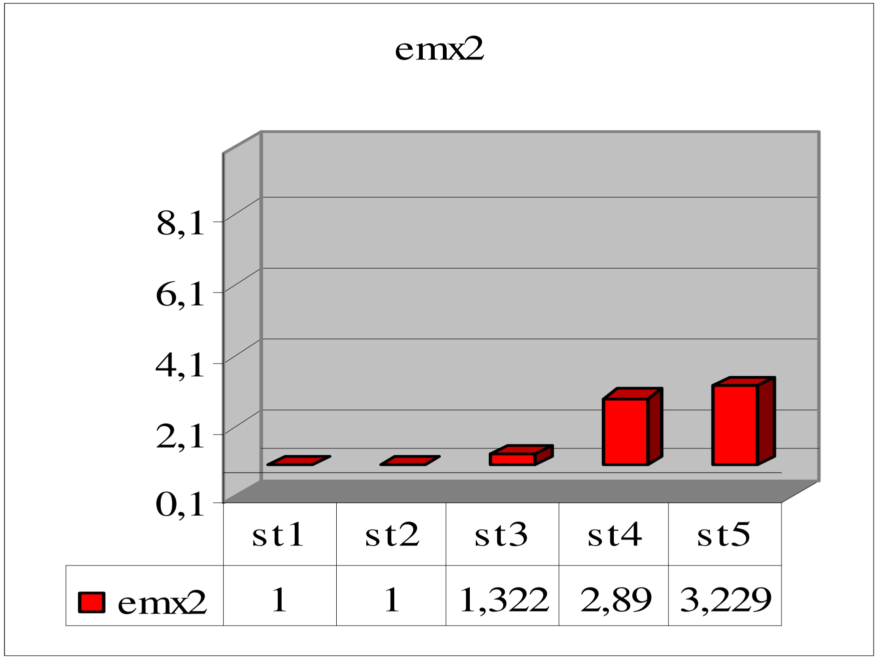
emx2
8,1
6,1
4,1
2,1
0,1
st1
st2
st3
st4
st5
emx2
1
1
1,322
2,89
3,229

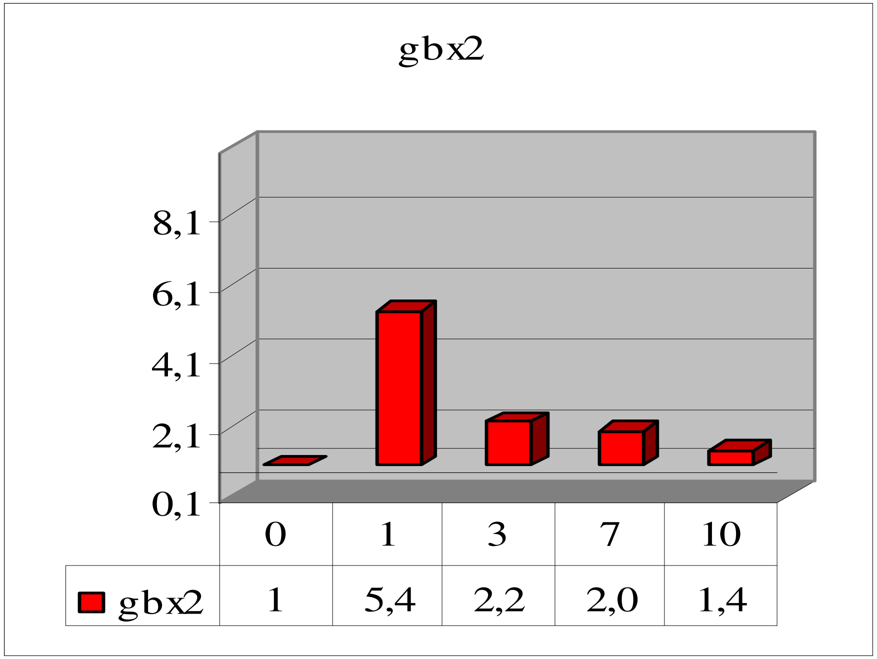
gbx2
8,1
6,1
4,1
2,1
0,1
0
1
3
7
10
gbx2
1
5,4
2,2
2,0
1,4

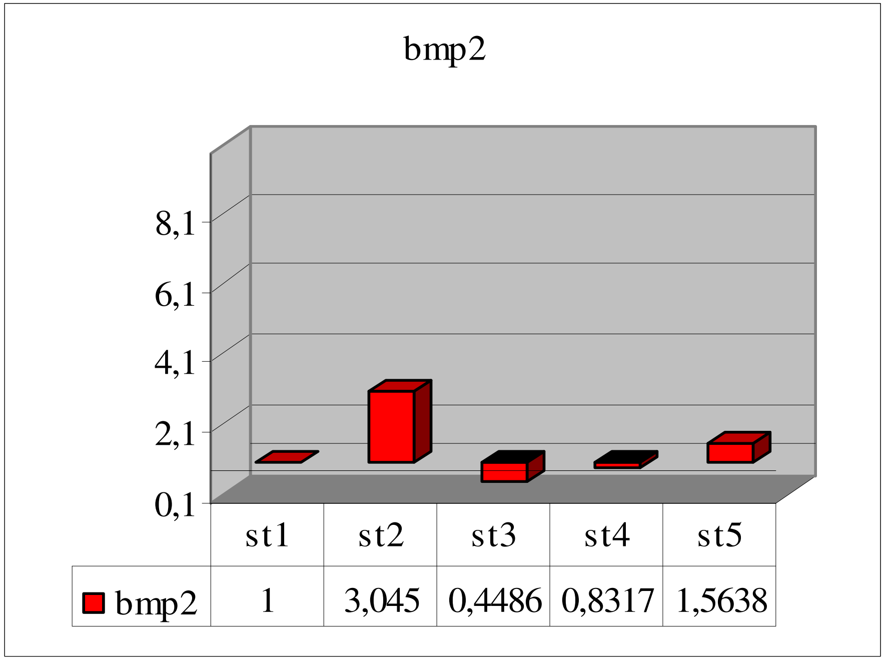

	st1	st2	st3	st4	st5
bmp2	1	3,045	0,4486	0,8317	1,5638

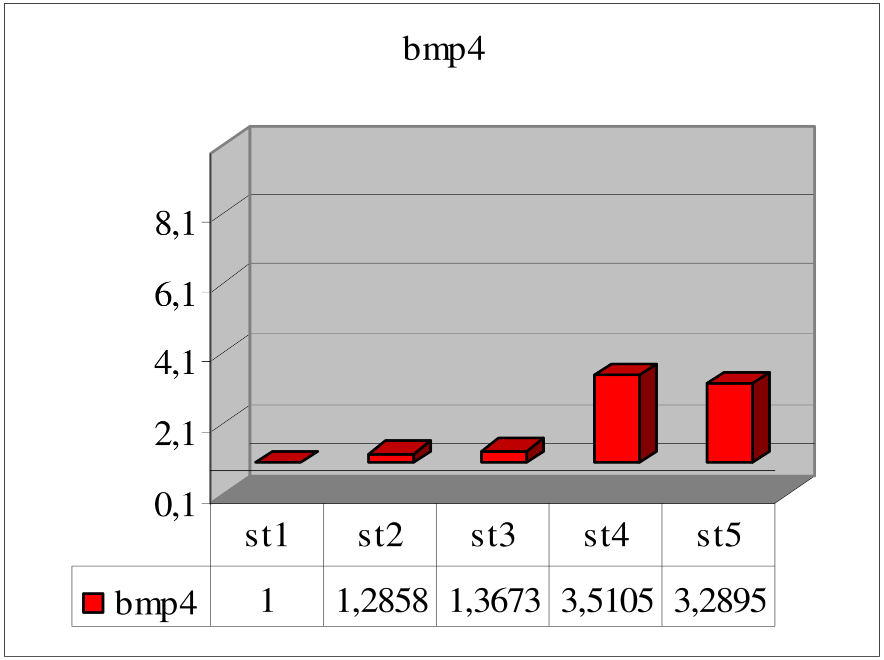

	st1	st2	st3	st4	st5
bmp4	1	1,2858	1,3673	3,5105	3,2895

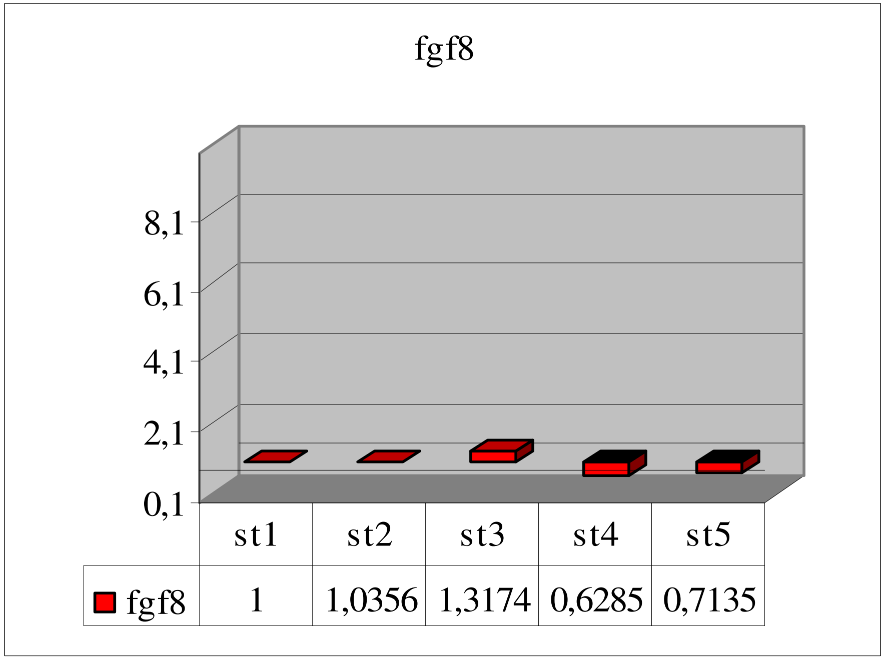

	st1	st2	st3	st4	st5
fgf8	1	1,0356	1,3174	0,6285	0,7135

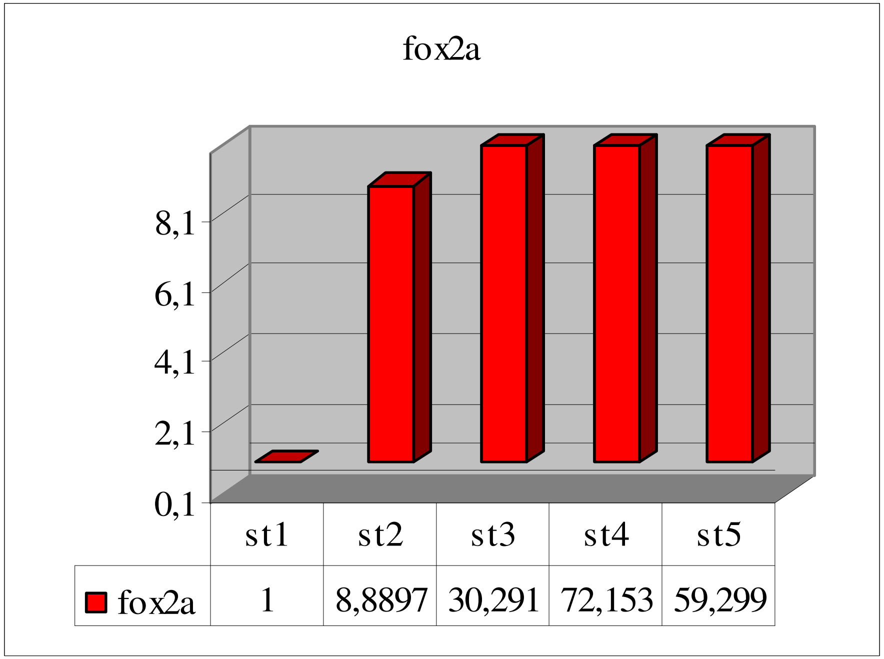

	st1	st2	st3	st4	st5
fox2a	1	8,8897	30,291	72,153	59,299

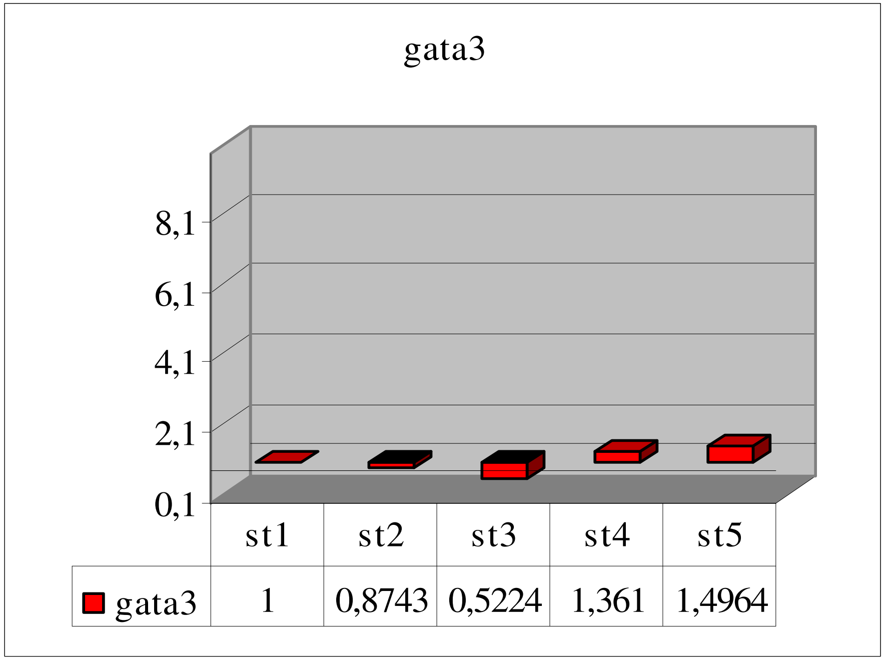
gata3
8,1
6,1
4,1
2,1
0,1
st1 st2 st3 st4 st5
gata3 1 0,8743 0,5224 1,361 1,4964

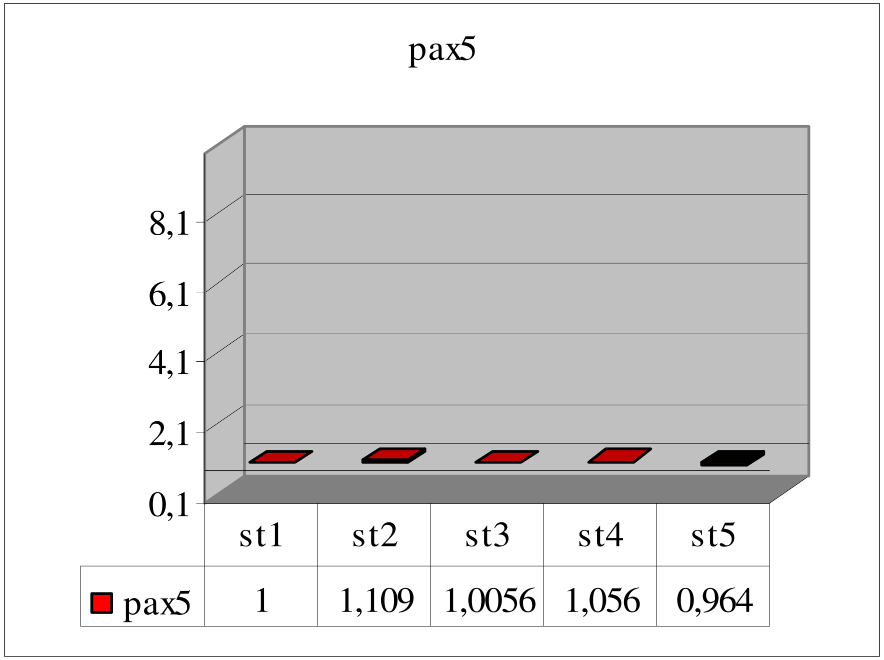
pax5
8,1
6,1
4,1
2,1
0,1
st1 st2 st3 st4 st5
pax5 1 1,109 1,0056 1,056 0,964

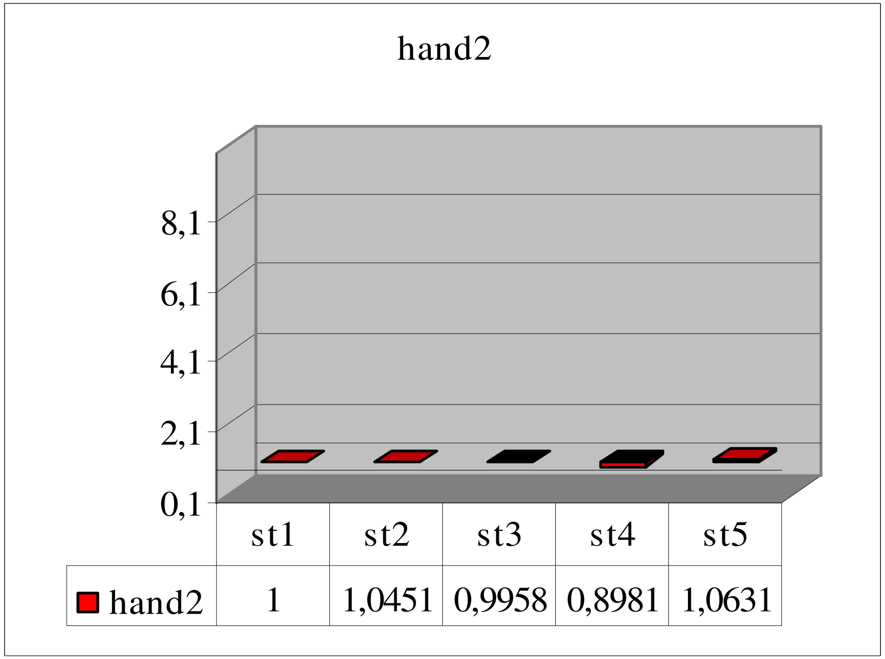
hand2
8,1
6,1
4,1
2,1
0,1
st1 st2 st3 st4 st5
hand2 1 1,0451 0,9958 0,8981 1,0631

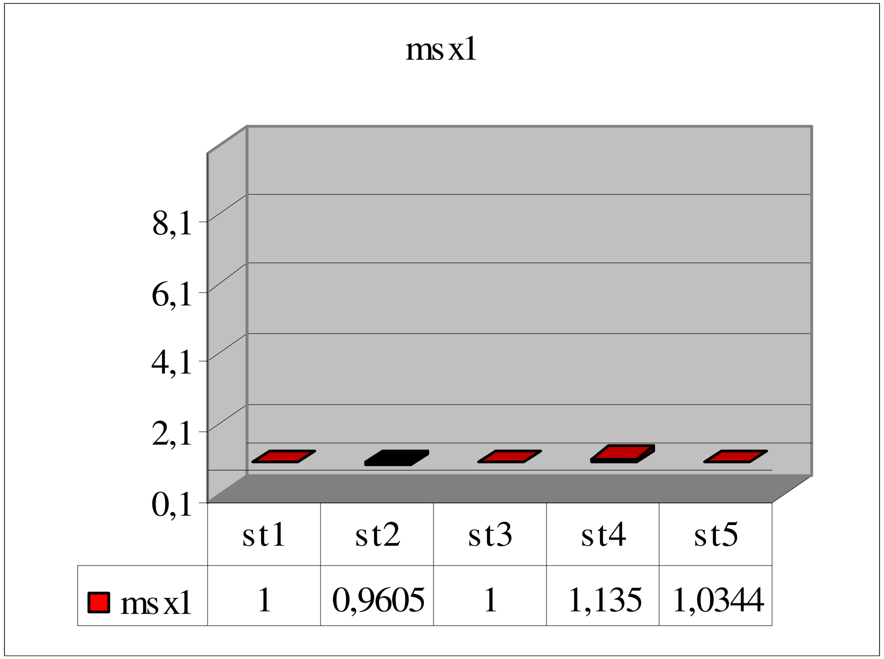
msx1
8,1
6,1
4,1
2,1
0,1
st1 st2 st3 st4 st5
msx1 1 0,9605 1 1,135 1,0344

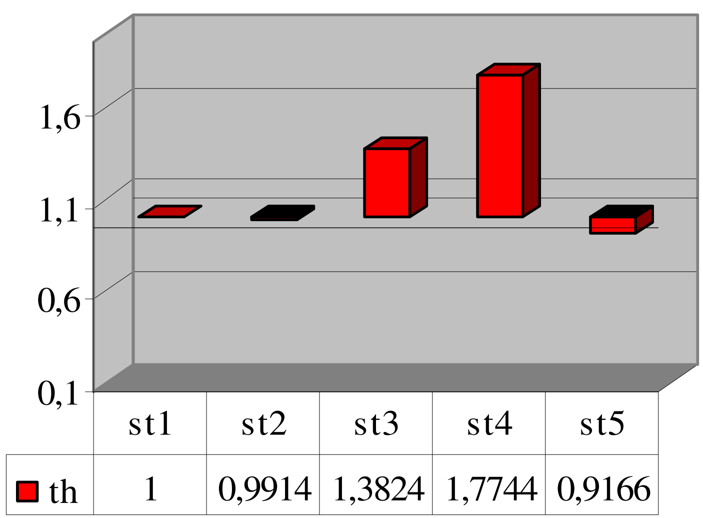

	st1	st2	st3	st4	st5
th	1	0,9914	1,3824	1,7744	0,9166

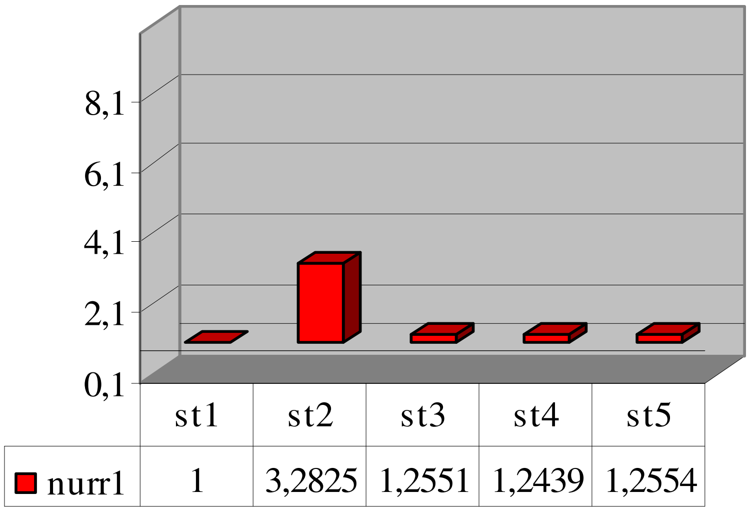

	st1	st2	st3	st4	st5
nurr1	1	3,2825	1,2551	1,2439	1,2554

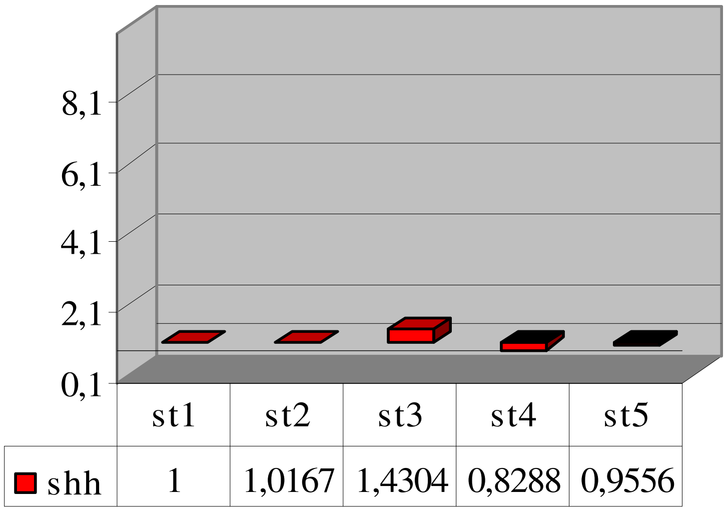

	st1	st2	st3	st4	st5
shh	1	1,0167	1,4304	0,8288	0,9556
